# Supplementary material for: Multi-classifier prediction of knee osteoarthritis progression from incomplete imbalanced longitudinal data
Source: Sci Rep. 2020 May 21;10:8427. doi: 10.1038/s41598-020-64643-8 (PMC7242357; doi:10.1038/s41598-020-64643-8)
Supplement: Supplementary file 1 — Supplementary Information. [file 41598_2020_64643_MOESM1_ESM.pdf]

Supplementary Information

Multi-classifier prediction of knee osteoarthritis progression from incomplete imbalanced longitudinal data

P. Widera, P.M.J. Welsing, C. Ladel, J. Loughlin, F.P.J.G. Lafeber, F. Petit Dop, J. Larkin, H. Weinans, A. Mobasheri, and J. Bacardit

	**33.3% size**		**66.7% size**		**100% size**	
	**median**	**95% CI**	**median**	**95% CI**	**median**	**95% CI**
**knn**	0.325	(0.308, 0.352)	0.329	(0.319, 0.350)	0.338	(0.327, 0.346)
**logreg**	0.361	(0.331, 0.370)	0.35	(0.337, 0.364)	0.364	(0.339, 0.377)
**logreg-multi**	0.355	(0.334, 0.367)	0.377	(0.348, 0.397)	0.389	(0.371, 0.418)
**random forest**	**0.399**	(0.378, 0.409)	**0.408**	(0.389, 0.427)	**0.425**	(0.411, 0.437)
**svc**	0.341	(0.306, 0.352)	0.341	(0.322, 0.362)	0.366	(0.336, 0.396)
**svc-rbf**	0.36	(0.318, 0.383)	0.361	(0.336, 0.379)	0.365	(0.350, 0.393)

Table S1. Comparison of algorithm performance on balanced subsets of the **CHECK** dataset (corresponding to FIGURE 1A). We report the median F1-score and confidence intervals around median (from binomial distribution) across all subsets and CV-repeats, for selected training set sizes (3/9, 6/9, and 9/9).

	**33.3% size**		**66.7% size**		**100% size**	
	**median**	**95% CI**	**median**	**95% CI**	**median**	**95% CI**
**knn**	0.369	(0.359, 0.379)	0.38	(0.378, 0.387)	0.389	(0.379, 0.396)
**logreg**	0.399	(0.377, 0.402)	0.415	(0.400, 0.421)	0.419	(0.412, 0.425)
**logreg-multi**	0.31	(0.298, 0.317)	0.323	(0.310, 0.339)	0.338	(0.322, 0.343)
**random forest**	**0.417**	(0.408, 0.431)	**0.43**	(0.425, 0.437)	**0.437**	(0.435, 0.443)
**svc**	0.36	(0.341, 0.371)	0.358	(0.351, 0.362)	0.375	(0.363, 0.386)
**svc-rbf**	0.41	(0.387, 0.419)	0.412	(0.398, 0.425)	0.426	(0.418, 0.435)

Table S2. Comparison of algorithm performance on balanced subsets of the **OAI** dataset (corresponding to FIGURE 1B). We report the median F1-score and confidence intervals around median (from binomial distribution) across all subsets and CV-repeats, for selected training set sizes (3/9, 6/9, and 9/9).

	**37.5% size**		**62.5% size**		**100% size**	
	**median**	**95% CI**	**median**	**95% CI**	**median**	**95% CI**
**1vsR**	0.491	(0.489, 0.491)	0.498	(0.497, 0.499)	0.502	(0.501, 0.502)
**duo**	**0.494**	(0.493, 0.495)	**0.504**	(0.503, 0.505)	**0.507**	(0.506, 0.508)
**multilabel**	0.489	(0.488, 0.489)	0.492	(0.491, 0.493)	0.496	(0.495, 0.497)
**single**	0.485	(0.484, 0.486)	0.49	(0.489, 0.491)	0.494	(0.493, 0.495)

Table S3. Comparison of performance of multi-model / multi-label methods trained on the **CHECK** dataset (corresponding to FIGURE 3A). We report the median F1-score and confidence intervals around median (from binomial distribution) across all CV-repeats, for selected training set sizes (3/8, 5/8, and 8/8).

	**42.9% size**		**71.4% size**		**100% size**	
	**median**	**95% CI**	**median**	**95% CI**	**median**	**95% CI**
**1vsR**	0.64	(0.640, 0.641)	0.641	(0.641, 0.641)	0.642	(0.642, 0.642)
**duo**	**0.644**	(0.643, 0.644)	**0.645**	(0.644, 0.645)	**0.646**	(0.646, 0.646)
**multilabel**	0.639	(0.638, 0.639)	0.639	(0.639, 0.639)	0.641	(0.641, 0.641)
**single**	0.638	(0.638, 0.639)	0.639	(0.639, 0.639)	0.64	(0.639, 0.640)

Table S4. Comparison of performance of multi-model / multi-label methods trained on the **OAI** dataset (corresponding to FIGURE 3B). We report the median F1-score and confidence intervals around median (from binomial distribution) across all CV-repeats, for selected training set sizes (3/7, 5/7, and 7/7).

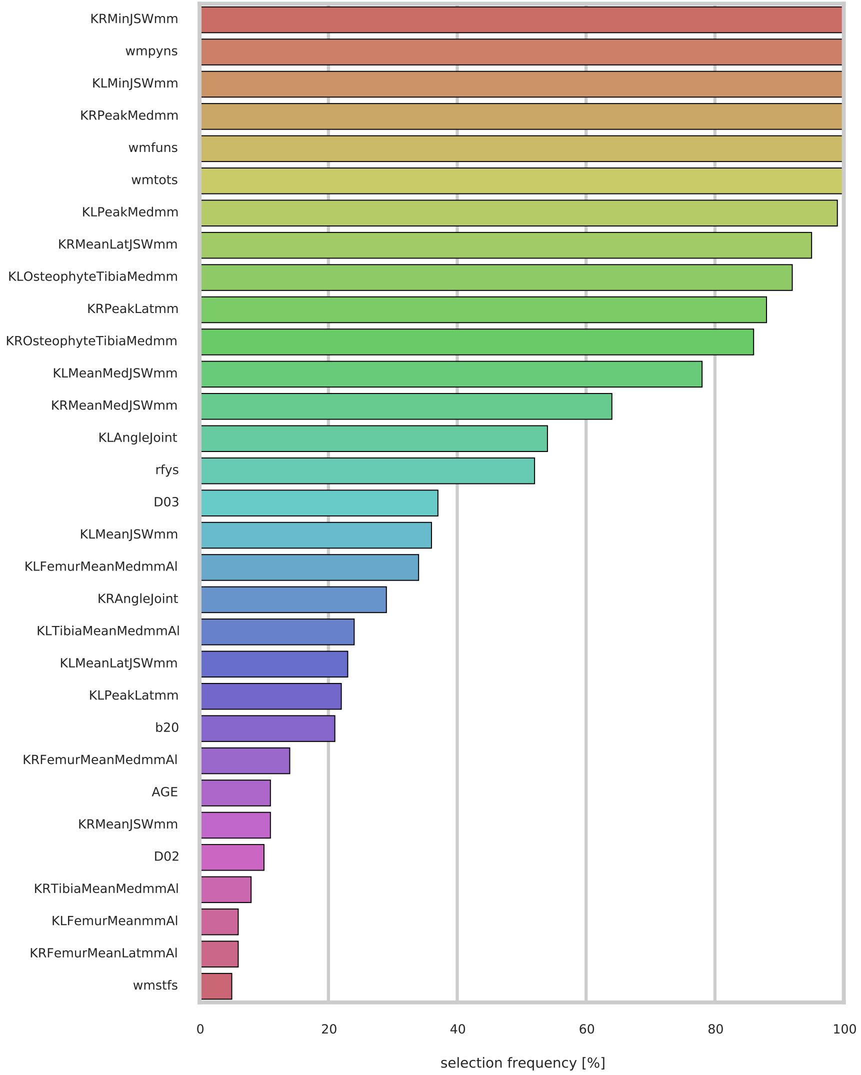

Figure S1. Frequency of feature selection with RFE procedure (**CHECK** dataset).

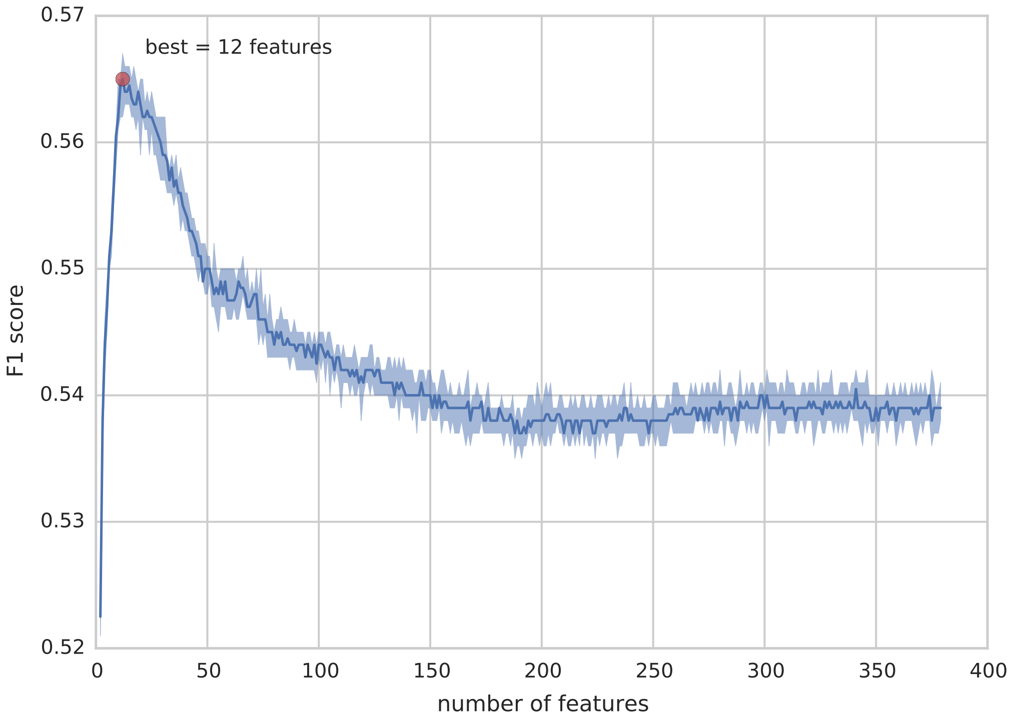

Figure S2. RFE scores (**CHECK** dataset).

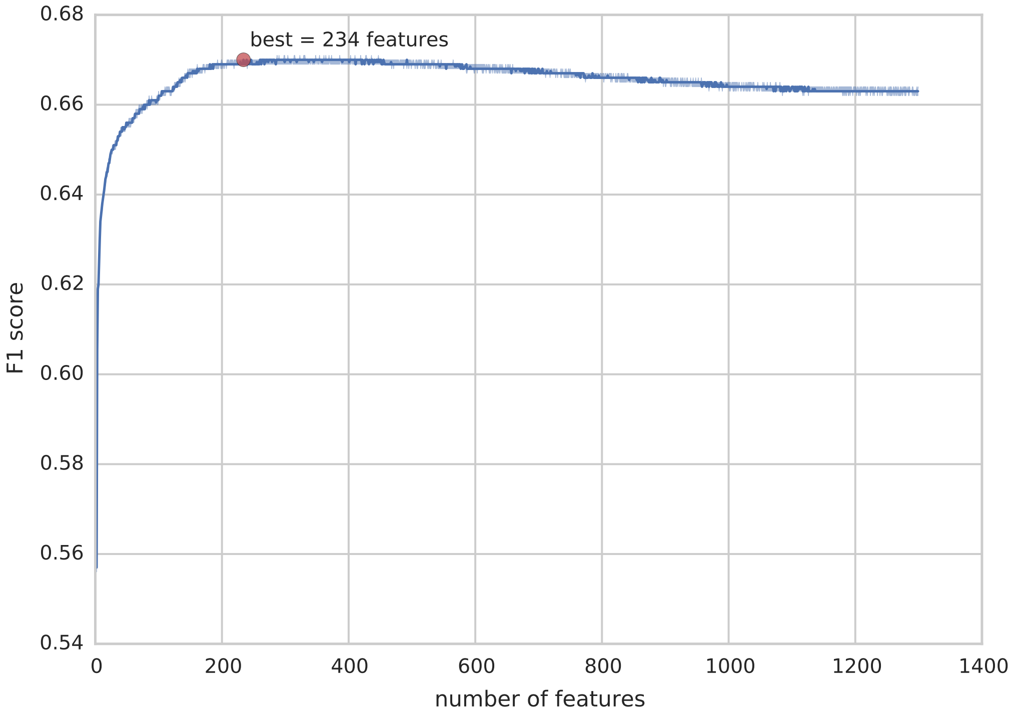

Figure S3. RFE scores (**OAI** dataset).

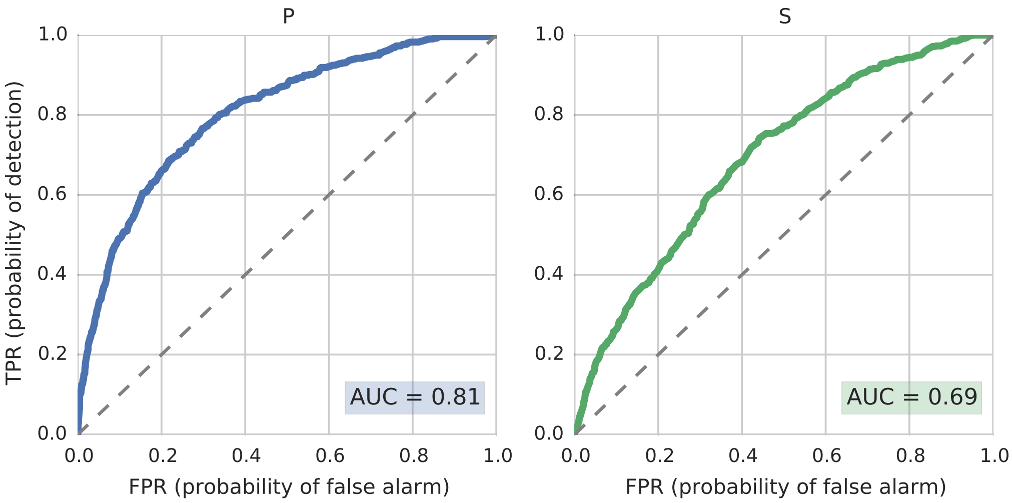

Figure S4. ROC curves for **P** and **S** sub-predictors of the best configuration of the *duo classifier* (**CHECK** dataset).

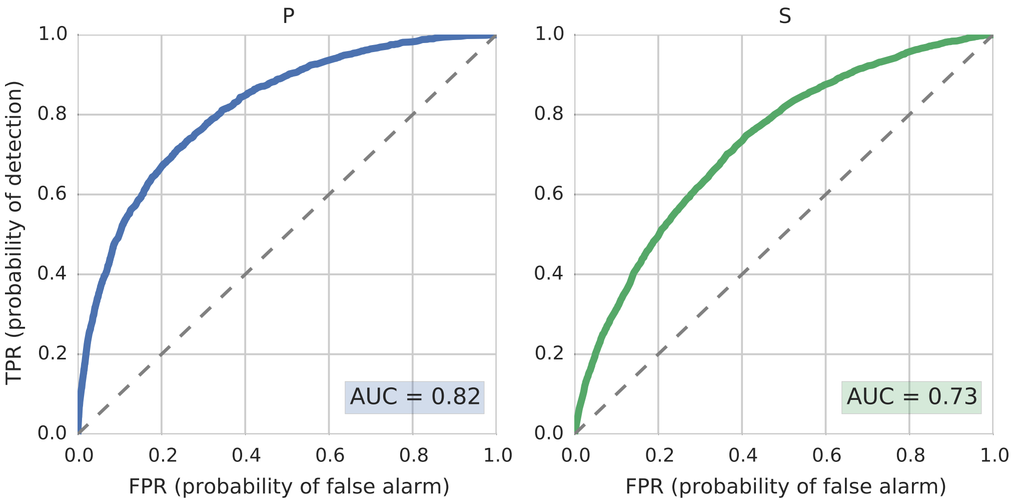

Figure S5. ROC curves for **P** and **S** sub-predictors of the best configuration of the *duo classifier* (**OAI** dataset).

(a) CHECK dataset

AGE	age
b20	how good is your health today
BEN	*undocumented* (EQ-5D health survey)
BMI	body mass index
D02	knee/hip pain intensity right know
D03	knee/hip pain intensity last week
geen	not using medication
HENDOL	left hip endorotation range of motion
HPIJN	hip pain
HSTIJF	morning stiffness in the hip
I09a	do you feel limited in your role as a partner
I09g	do you feel limited in fulfilling volunteering work
KFLEXL	left knee flexion range of motion
KLAngleJoint	left knee angle between the femur and tibia
KLFemurMeanMedmmAl	left knee mean medial femur bone density
KLMeanMedJSWmm	left knee mean medial joint space width
KLMinJSWmm	left knee minimum total joint space width
KLOsteophyteTibiaLatmm	left knee lateral tibia osteophyte area
KLOsteophyteTibiaMedmm	left knee medial lateral tibia osteophyte area
KLTibiaMeanMedmmAl	left knee mean medial tibia bone density
KPIJN	knee pain
KRAngleJoint	right knee angle between the femur and tibia
KRMeanMedJSWmm	right knee mean medial joint space width
KRMinJSWmm	right knee minimum total joint space width
KROsteophyteTibiaMedmm	right knee medial lateral tibia osteophyte area
KRPeakMedmm	right knee medial tibial eminence height
KSTIJF	morning stiffness in the knee
mobility	mobility (EQ-5D health survey)
MVH_A1	index based on MVH-A1 value set (EQ-5D health survey)
pain	pain or discomfort (EQ-5D health survey)
PCIpiekr	worring (Pain Coping Inventory)
rfys	physical functioning (SF-36 health survey)
rment	general mental health (SF-36 health survey)
rpijn	bodily pain (SF-36 health survey)
rvit	vitality (SF-36 health survey)
wmfuns	physical functioning sub-score (WOMAC)
wmpyns	pain sub-score (WOMAC)
wmstfs	stiffness sub-score (WOMAC)
wmtots	total score (WOMAC)

(b) OAI dataset

DFBCOLL	difference in minutes between baseline and follow-up blood collection times
DFUCOLL	difference in minutes between baseline and follow-up urine collection times
DILKN16	difficulty of heavy chores in last week (WOMAC)
DIRKN12	difficulty of lying down in teh last 7 days (WOMAC)
DIRKN6	pain level while walking in the last 7 days (WOMAC)
GLCFQCV	glucosamine frequency of use in past 6 months
HSPSS	physical summary score (SF-12 health survey)
KGLRS	how much the knee pain and arthritis affect you?
KIKBALL_3.0	leg used to kick a bal
KOOSKPL	left knee pain score (KOOS)
KOOSKPR	right knee pain score (KOOS)
KOOSQOL	quality of life score (KOOS)
KOOSYMR	symptoms score (KOOS)
KPRKN2	pain while fully straightening the knee in the last 7 days (KOOS)
P7LKACV	average left knee pain in the last 7 days
P7LKRCV	left knee pain severity in the last 7 days
P7RKACV	right knee pain severity in the last 7 days
P7RKRCV	average right knee pain in the last 7 days
PMLKRCV	left knee pain severity in the last 30 days
PMRKRCV	right knee pain severity in the last 30 days
S1_CFWDTH	width of femoral condyles used to define x = 1.0
S1_IMPIXSZ	pixel size used for convertion to millimetres
S1_JSW150	medial JSW at x = 0.15mm
S1_JSW175	medial JSW at x = 0.175mm
S1_JSW200	medial JSW at x = 0.2mm
S1_MCAJSW	average medial joint space width
S1_MCMJSW	minimum medial joint space width
S1_TMJSW	total minimum joint space width
S1_TPCFDS	distance from tibial plateau to tibial rim closest to femoral condyle
S2_IMPIXSZ	pixel size used for convertion to millimetres
S2_JSW150	medial JSW at x = 0.15mm
S2_JSW175	medial JSW at x = 0.175mm
WOMADLL	left knee disability score (WOMAC)
WOMADLR	right knee disability score (WOMAC)
WOMKPL	left knee pain score (WOMAC)
WOMKPR	right knee pain score (WOMAC)
WOMTSL	left knee total score (WOMAC)
WOMTSR	right knee total score (WOMAC)
WPLKN3	knee pain at night while in bed in the last 7 days
WPLKN4	knee pain sitting or lying down in the last 7 days

Table S5. Description of attributes shown in impact plots (FIGURES 6 to 9).

List of attributes used in model training (after preprocessing)

CHECK

A03, ADDUC, ADDUCL, ADDUCR, AGE, B18a, BAKERHK, BAKERKL, BAKERKR, BAKERLR, BAND, BANDHK, BANDKL, BANDKR, BANDLR, BEN, BMI, BOU2L, BOU2R, BOU3L, BOU3R, BOU4L, BOU4R, BOU5L, BOU5R, D01a, D02, D03, F01_ha, HABDL, HABDPIJNL, HABDPIJNR, HABDR, HABDSTRTL, HABDSTRTR, HADDL, HADDPIJNL, HADDPIJNR, HADDR, HADDSTRTL, HADDSTRTR, HCONTRACL, HCONTRACR, HEB2L, HEB2R, HEB3L, HEB3R, HEB4L, HEB4R, HEB5L, HEB5R, HENDOL, HENDOPIJNL, HENDOPIJNR, HENDOR, HENDOSTRTL, HENDOSTRTR, HEXOL, HEXOPIJNL, HEXOPIJNR, HEXOR, HEXOSTRTL, HEXOSTRTR, HFLEXL, HFLEXPIJNL, HFLEXPIJNR, HFLEXR, HFLEXSTRTL, HFLEXSTRTR, HPIJN, HPIJNL, HPIJNR, HSTIJF, I09a, I09b, I09c, I09d, I09e, I09f, I09g, I09h, I09i, I10, I13a, I13b, I13c, I13d, I13e, I13f, I13g, I13h, I13i, I13j, I14, ILIO, ILIOL, ILIOR, INFRACT, INFRACTH, INFRACTHKR, ITISRF, J01, J02a, J02b, J02c, J03, J04a, J04b, J04c, J04d, J04e, J04f, J04g, J04h, J04i, J04j, J04k, J04m, J05a, J05b, J05c, J05d, J05e, J06A, J06B, J06C, J06D, J06E, J06F, J06G, KBENIGL, KBENIGR, KCREPL, KCREPR, KDRUKPL, KDRUKPR, KEXTPIJNL, KEXTPIJNR, KEXTROML, KEXTROMR, KFLEXL, KFLEXPIJNL, KFLEXPIJNR, KFLEXR, KLAngleJoint, KLFemurMeanLatmmAl, KLFemurMeanMedmmAl, KLFemurMeanmmAl, KLKL, KLLatJSN, KLMeanJSWmm, KLMeanLatJSWmm, KLMeanMedJSWmm, KLMedJSN, KLMinJSWmm, KLOsteophyteFemurLatmm, KLOsteophyteFemurMedmm, KLOsteophyteTibiaLatmm, KLOsteophyteTibiaMedmm, KLPeakLatmm, KLPeakMedmm, KLTibiaMeanLatmmAl, KLTibiaMeanMedmmAl, KLTibiaMeanmmAl, KPATGRL, KPATGRR, KPIJN, KPIJNL, KPIJNR, KRAngleJoint, KREFILLL, KREFILLR, KRFemurMeanLatmmAl, KRFemurMeanMedmmAl, KRFemurMeanmmAl, KRKL, KRLatJSN, KRMeanJSWmm, KRMeanLatJSWmm, KRMeanMedJSWmm, KRMedJSN, KRMinJSWmm, KROsteophyteFemurLatmm, KROsteophyteFemurMedmm, KROsteophyteTibiaLatmm, KROsteophyteTibiaMedmm, KRPeakLatmm, KRPeakMedmm, KRTibiaMeanLatmmAl, KRTibiaMeanMedmmAl, KRTibiaMeanmmAl, KSTIJF, KUITGL, KUITGR, KWARMTEL, KWARMTER, MVH_A1, Missos, PCIaflei, PCIeisen, PCIpiekr, PCIpijn, PCIrust, PCIterug, QUAD, QUADL, QUADR, RADIC, RAS, SEPART, SEPARTHKR, SEPARTK, SEXE, SLR, SLRL, SLRR, Sosfrec, Sosgem, Sosirec, Soskrec, TROCH, TROCHL, TROCHR, V18A, V18B, V18C, V18D, V18E, V18F, V18G, V18J, V18K, V18L, V18M, V18N, V18O, V18Q, V18R, V18S, V18T, V18U, V18V, V18W, V18X, V18Y, VISITNR, ZW01, ZW02, ZW03, ZW05agr, ZW05amb, ZW05tra, ZW07a, ZW07b, ZW07c, ZW07d, ZW07e, ZW07f, ZW07g, ZW3a, a08, anders, asp, b20, baker, comor, d01b, d01c, d01d, d01e, daily, f01_andh, f01_ands, f01_bedr, f01_ergo, f01_fys, f01_geen, f01_gmd, f01_gzh, f01_mw, f01_oefth, f01_orth, f01_past, f01_psy, f01_reu, f01_rev, f01_wvk, geen, ibu, mobility, mood, pain, parac, rfys, rgezv, rment, rpijn, rrolem, rrolfy, rsoc, rvit, selfcare, soscrec, sostotal, study_group, wmfuns, wmpyns, wmstfs, wmtots, zw05adm, zw05and, zw05comm, zw05dnst, zw08adm, zw08agr, zw08amb, zw08and, zw08comm, zw08dnst, zw08tra, zw3b, zw3c, zw3d, zw3e, zw3f, zw3g.

OAI

20MPACE, 400EXCL, 400MCMP, ABCIRC, ACUSCV, ACUTCV, AGE, ARTDOC, ARTDRCV, ARTH12, ASTHMA, BISPHOS, BISPTYP, BLDCOLL, BLDHRS1, BLDRAW1, BLUPMN1, BMI, BMICAT, BONFX, BP30, BP30OFT, BPACTCV, BPARM, BPBAD, BPBEDCV, BPCFSZ, BPDAYCV, BPDIAS, BPSYS, BPTOT, BRAC12, BRACCV, BYPLEG, CAM12, CANCER, CAPSNCV, CEMPLOY, CESD, CESD1, CESD10, CESD11, CESD12, CESD13, CESD14, CESD15, CESD16, CESD17, CESD18, CESD19, CESD2, CESD20, CESD3, CESD4, CESD5, CESD6, CESD7, CESD8, CESD9, CHIRCV, CHNFQCV, CHON, CITRATE, COMORB, COMP10, COXIBS, CS5, CSPACE, CSTIME1, CSTIME2, CSTREP1, CSTREP2, CSTSGL, CUREMP, DFBCOLL, DFUCOLL, DIAB, DIASELG, DIET12, DIETCV, DILKN1, DILKN10, DILKN11, DILKN12, DILKN13, DILKN14, DILKN15, DILKN16, DILKN17, DILKN2, DILKN3, DILKN4, DILKN5, DILKN6, DILKN7, DILKN8, DILKN9, DIRKN1, DIRKN10, DIRKN11, DIRKN12, DIRKN13, DIRKN14, DIRKN15, DIRKN16, DIRKN17, DIRKN2, DILKN8, DILKN9, DIRKN1, DIRKN10, DIRKN11, DIRKN12, DIRKN13, DIRKN14, DIRKN15, DIRKN16, DIRKN17, DIRKN2, DIRKN3, DIRKN4, DIRKN5, DIRKN6, DIRKN7, DIRKN8, DIRKN9, DOXYCYC, EDTA, ESTR, EXCESS1, FALL, FALLCV, GLCFQCV, GLUC, GNRH, GT12CV, HEMAT1, HERB12, HERBCV, HOSPSUR, HOUACT1, HOUACT2, HOUACT3, HOUACT4, HOUACT5, HOUACT6, HOURSP1, HOURWK, HPL12CV, HPNL12, HPNR12, HPR12CV, HRELG, HRS12, HRSUC1, HRTAT, HRTFAIL, HSMSS, HSPSS, HYAINJL, HYAINJR, HYINJCV, ID, ILLPWK1, INJL12, INJR12, ISOREQ, KEXAMK, KGLRS, KIDFXN, KIKBALL, KNINJ, KOOSFSR, KOOSFX1, KOOSFX4, KOOSFX5, KOOSKPL, KOOSKPR, KOOSQOL, KOOSYML, KOOSYMR, KPA30CV, KPACDCV, KPACT30, KPACTCV, KPL12CV, KPL30CV, KPLK20B, KPLK20D, KPLKN1, KPLKN2, KPLKN3, KPMED, KPMEDCV, KPNL12, KPNR12, KPR12CV, KPR30CV, KPRK20B, KPRK20D, KPRKN1, KPRKN2, KPRKN3, KQOL1, KQOL2, KQOL3, KQOL4, KSRGL12, KSRGR12, KSX, KSXLKN1, KSXLKN2, KSXLKN3, KSXLKN4, KSXLKN5, KSXRKN1, KSXRKN2, KSXRKN3, KSXRKN4, KSXRKN5, LEAKAG1, LEXCOMP, LEXP1CV, LEXP2CV, LEXPN, LFXCOMP, LFXP1CV, LFXP2CV, LFXPN, LIVDAM, LKABPN, LKALNMT, LKEFFB, LKEFFPT, LKLTTPN, LKMTTPN, LKP30CV, LKPFCRE, LKPGDPN, LKSX, LLWGT, LUNG, MASSCV, MAXCIRC, MISSWK, MRITEM3, MRSEQNL, MRSEQNR, MSM, MULTST1, NARCOT, NSAIDRX, NSAIDS, OAB12CV, OAD12CV, OAF12CV, OAH12CV, OAO12CV, OTA12CV, OTHVP1, OTREASM, P30VITC, P30VITD, P30VITE, P30VT1, P30VT10, P30VT2, P30VT3, P30VT4, P30VT5, P30VT6, P30VT7, P30VT8, P30VT9, P7LKACV, P7LKFR, P7LKRCV, P7RKACV, P7RKFR, P7RKRCV, PA130, PA130CV, PA130NF, PA230, PA230CV, PA330, PA330CV, PA430, PA430CV, PA530, PA530CV, PASE, PASE1, PASE1HR, PASE2, PASE2HR, PASE3, PASE4, PASE5, PASE6, PLAQHR1, PMLKRCV, PMRKRCV, PNMEDT, POLYRH, PTH, QOVP1, RA, RAIA12, RASTAFU, RELA12, RELACV, REMITM, REXCOMP, REXP1CV, REXP2CV, REXPN, RFXCOMP, RFXP1CV, RFXP2CV, RFXPN, RKABPN, RKALNMT, RKEFFB, RKEFFPT, RKLTTPN, RKMTTPN, RKP30CV, RKPFCRE, RKPGDPN, RKSX, RLARM, RLBACK, RLHORIZ, RLLGTH, RLVERT, RLWGT, RPAVG, RUBCV, RUBS12, RX30, RX30NUM, RXACTM, RXANALG, RXASPRN, RXBISPH, RXCHOND, RXCLCTN, RXCLCXB, RXCOX2, RXFLUOR, RXGLCSM, RXIHYAL, RXISTRD, RXMSM, RXNARC, RXNSAID, RXNTRAT, RXOSTRD, RXOTHAN, RXRALOX, RXRFCXB, RXSALIC, RXTPRTD, RXVIT_D, RXVLCXB, S1_CFWDTH, S1_IMPIXSZ, S1_INCPLL, S1_INCPLM, S1_INCSTPS, S1_JSW150, S1_JSW175, S1_JSW200, S1_JSW225, S1_JSW250, S1_JSW275, S1_JSW300, S1_LCAJSW, S1_LCMJSW, S1_LJSW700, S1_LJSW725, S1_LJSW750, S1_LJSW775, S1_LJSW800, S1_LJSW825, S1_LJSW850, S1_LJSW875, S1_LJSW900, S1_LTPMEBE, S1_MCAJSW, S1_MCMJSW, S1_MJSWBB, S1_NOLJSWX, S1_NOLMIN, S1_NOMJSWX, S1_NOMMJSW, S1_TAJSW, S1_TMJSW, S1_TPCFDS, S1_XMJSW, S1_XRATTL, S1_XRATTM, S1_XRCHL, S1_XRCHM, S1_XRCYFL, S1_XRCYFM, S1_XRCYTL, S1_XRCYTM, S1_XRJSL, S1_XRJSM, S1_XRKL, S1_XROSFL, S1_XROSFM, S1_XROSTL, S1_XROSTM, S1_XRSCFL, S1_XRSCFM, S1_XRSCTL, S1_XRSCTM, S2_CFWDTH, S2_IMPIXSZ, S2_INCPLL, S2_INCPLM, S2_INCSTPS, S2_JSW150, S2_JSW175, S2_JSW200, S2_JSW225, S2_JSW250, S2_JSW275, S2_JSW300, S2_LCAJSW, S2_LCMJSW, S2_LJSW700, S2_LJSW725, S2_LJSW750, S2_LJSW775, S2_LJSW800, S2_LJSW825, S2_LJSW850, S2_LJSW875, S2_LJSW900, S2_LTPMEBE, S2_MCAJSW, S2_MCMJSW, S2_MJSWBB, S2_NOLJSWX, S2_NOLMIN, S2_NOMJSWX, S2_NOMMJSW, S2_TAJSW, S2_TMJSW, S2_TPCFDS, S2_XMJSW, S2_XRATTL, S2_XRATTM, S2_XRCHL, S2_XRCHM, S2_XRCYFL, S2_XRCYFM, S2_XRCYTL, S2_XRCYTM, S2_XRJSL, S2_XRJSM, S2_XRKL, S2_XROSFL, S2_XROSFM, S2_XROSTL, S2_XROSTM, S2_XRSCFL, S2_XRSCFM, S2_XRSCTL, S2_XRSCTM, SAME, SEAQHR1, SERUM, SF1, SF10, SF11, SF12, SF2, SF3, SF4, SF5, SF6, SF7, SF8, SF9, SMRIELG, SPIR12, SPIRCV, SREPHR, SREPLKR, SREPRKR, SRGSTAT, STEPST1, STEPST2, STINJCV, STRINJL, STRINJR, STROKE, SYSELG, TIMET1, TIMET2, TRANSET, TYLEN, ULCER, URINHR1, URINOB1, URNCOLL, URUPMN1, VCOLL1, VEIN1, VITM12, VITMCV, VOID1, W20COMP, WALKER, WEEKWK, WEIGHT, WKHR7CV, WLK20T1, WLK20T2, WLKAID, WOMADLL, WOMADLR, WOMKPL, WOMKPR, WOMSTFL, WOMSTFR, WOMTSL, WOMTSR, WORK7, WORKAMT, WPLKN1, WPLKN2, WPLKN3, WPLKN4, WPLKN5, WPRKN1, WPRKN2, WPRKN3, WPRKN4, WPRKN5, WSLKN1, WSLKN2, WSRKN1, WSRKN2, YOGA12, YOGACV.
